# CDK4/6 Inhibitors—Overcoming Endocrine Resistance Is the Standard in Patients with Hormone Receptor-Positive Breast Cancer

**DOI:** 10.3390/cancers15061763

**Published:** 2023-03-14

**Authors:** Naiba Nabieva, Peter A. Fasching

**Affiliations:** 1 Department of Gynecology and Obstetrics, Erlangen University Hospital, Comprehensive Cancer Center Erlangen-EMN, Friedrich-Alexander Universität Erlangen-Nürnberg, 91054 Erlangen, Germany; naiba.nabieva@fau.de; 2 GynPraxis Dr. Ernst and Colleagues, 91054 Erlangen, Germany

**Keywords:** breast cancer, endocrine treatment, CDK4/6 inhibitor, abemaciclib, dalpiciclib, palbociclib, ribociclib

## Abstract

**Simple Summary:**

Abemaciclib, dalpiciclib, palbociclib and ribociclib have all demonstrated significant improvements in progression-free survival in advanced disease. However, to date, abemaciclib and ribociclib are the only CDK4/6 inhibitors shown to improve the overall survival in patients with metastatic breast cancer. Moreover, abemaciclib is the first CDK4/6 inhibitor to also reduce the risk of recurrence in those with early-stage disease. Thus, achieving significant improvements in survival rates in the advanced and early breast cancer treatment setting, CDK4/6 inhibitors are the first substances in almost two decades to substantially change the standard of care for advanced breast cancer patients. This review is designed to discuss the recent history, current role, future directions and opportunities of this substance class.

**Abstract:**

Purpose of review: Tamoxifen and aromatase inhibitors can be considered as some of the first targeted therapies. For the past 30 years, they were the endocrine treatment standard in the advanced and early breast cancer setting. CDK4/6 inhibitors, however, are the first substances in almost two decades to broadly improve the therapeutic landscape of hormone receptor-positive breast cancer patients for the upcoming years. This review is designed to discuss the recent history, current role, future directions and opportunities of this substance class. Recent findings: The CDK4/6 inhibitors abemaciclib, dalpiciclib, palbociclib and ribociclib have all demonstrated a statistically significant improvement in progression-free survival in advanced disease. However, to date, abemaciclib and ribociclib are the only CDK4/6 inhibitors to have shown an improvement in overall survival in patients with metastatic breast cancer. Moreover, abemaciclib is the first CDK4/6 inhibitor to also reduce the risk of recurrence in those with early-stage disease. Further CDK inhibitors, treatment combinations with other drugs and different therapy sequences are in development. Summary: Achieving significant improvements in survival rates in the advanced and early breast cancer treatment setting, CDK4/6 inhibitors have set a new standard of care for patients with advanced breast cancer. It remains important to better understand resistance mechanisms to be able to develop novel substances and treatment sequences.

## 1. Introduction

The development of endocrine treatment (ET) for breast cancer (BC) patients started at the end of the 19th century when Sir George Thomas Beatson found out that a bilateral oophorectomy results in an improvement in advanced breast cancer (aBC) lesions [1]. However, the discovery and investigation of drugs targeting the hormone receptor took almost 80 years. Thus, in the 1970s, with tamoxifen as a selective estrogen receptor modulator (SERM), the first target therapy was approved for the treatment of hormone receptor-positive BC patients [2]. Two decades later, in the 1990s, a group of further substances—the aromatase inhibitors (AIs) anastrozole, exemestane and letrozole—received approval status as, compared to tamoxifen, they improved the outcome of postmenopausal women with aBC [3,4]. These were followed soon by the approval of fulvestrant in 2002, a selective estrogen receptor degrader (SERD) that led to a longer duration of response than anastrozole in postmenopausal patients [5]. Due to positive study results, all of the above-mentioned therapeutics but fulvestrant reached the treatment setting of non-advanced BC [6,7]. Therefore, being successful in the therapy of advanced as well as early breast cancer (eBC) patients, tamoxifen and AIs have set the ET standard for the past 30 years and were later only complemented by potential additional ovarian function suppression (OFS) with a gonadotropin-releasing hormone agonist to further reduce hormone blood levels in premenopausal women [8].

**Figure 1:**
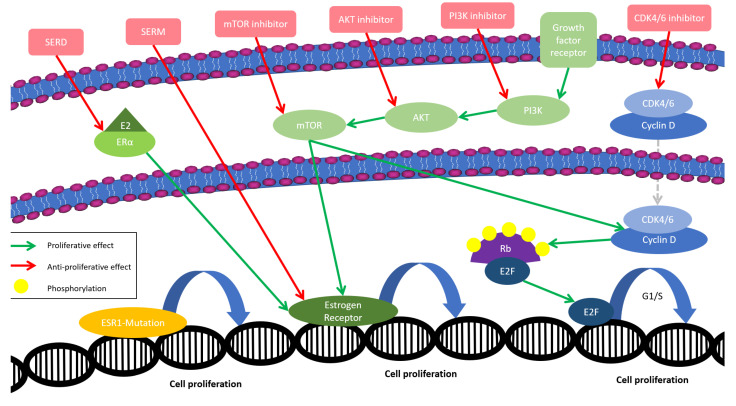
Pathways potentially contributing to endocrine treatment resistance (simplified representation) [14,15]. AKT: AKT murine thymoma viral oncogene; CDK4/6: cyclin-dependent kinase 4/6; E2: estradiol; E2F: transcription factor; ERalpha: estrogen receptor alpha; ESR1: estrogen receptor 1 gene; mTOR: mammalian target of rapamycin; PI3K: phosphatidylinositol 3-kinase; Rb: retinoblastoma protein; SERD: selective estrogen receptor degrader; SERM: selective estrogen receptor modulator. In HR-positive BC cells, different mechanisms may lead to hyperactivation of the cyclin D-CDK4/6-retinoblastoma pathway. The activation of the PI3K-AKT-mTOR pathway can increase cyclin D levels or enhance its activity through post-translational mechanisms. Moreover, in contrast to triple-negative BC cells, where Rb is mostly absent or dysfunctional, in HR-positive BC cells, it is usually retained. Genomic factors, which encode for the endogenous inhibition of the CDK4/6 or are involved in the transcription of the estrogen receptor, explain why CDK4/6 plays a significant role in HR-positive BC in special. Regarding resistance mechanisms, mutations (e.g., *FAT1*) and a loss of functional Rb in particular are discussed to be associated with de novo and acquired CDK4/6i resistance [16].

The introduction of everolimus represents a milestone in the treatment of hormone receptor-positive, HER2-negative BC patients. For the first time, endocrine resistance could be overcome for patients with advanced disease [9]. Furthermore, Alpelisib was the second therapy to show that endocrine resistance could be overcome in patients with *PIK3CA*-mutated hormone receptor-positive, HER2-negative aBC [10]. However, everolimus did not achieve an improvement in outcomes in the early therapy setting [11]. Figure 1 shows the diverse pathways within the cell cycle that are potential contributors to ET resistance.

Inhibitors of the cyclin-dependent kinase 4/6 (CDK4/6i) are the first substances in almost two decades to be effective in both advanced and early BC patients. Having improved survival outcomes in stage IV disease first and being later additionally successful in the therapy of stage II and III BC, CDK4/6i in combination with ET substantially improved the therapeutic landscape of hormone receptor-positive disease and became the new standard of care [12,13].

## 2. The Early Development of CDK4/6 Inhibitors in Patients with Hormone Receptor-Positive, HER2-Negative Advanced Breast Cancer

### 2.1. Impact on Progression-Free Survival

The first CDK4/6i to be tested in human breast cancer cell lines was palbociclib (Ibrance^®^, PD-0332991, Pfizer, New York, NY, USA). It showed an effect, especially in hormone receptor-positive and HER2-amplified cell lines, which was the reason for its further development [17]. In two randomized phase III trials, it later demonstrated a statistically significant prolongation of progression-free survival (PFS), changing the standard-of-care treatment of women with hormone receptor-positive, HER2-negative aBC [18,19]. Soon after, two other substances inhibiting the CDK4/6 could show similar results. In several trials, abemaciclib (Verzenio^®^, LY2835219, Lilly, Indianapolis, IN, USA) and ribociclib (Kisqali^®^, LEE011, Novartis, Basel, Switzerland) also significantly improved the PFS of women suffering from aBC [20,21,22,23,24]. Later, dalpiciclib (Ai Rui Kang, SHR6390, Jiangsu Hengrui, Lianyungang, Jiangsu Province, China)—a fourth drug from this family—could also show a benefit with regard to PFS [25,26]. In some of those trials, especially in the first line, patients using CDK4/6i were free from disease progression for up to 30 months (Table 1).

### 2.2. Improvement in Overall Survival

Despite a partly diverse side effect profile between the substances, the positive PFS results throughout all trials implied a CDK4/6i class effect. This was questioned when overall survival (OS) outcomes in the advanced setting and outcome differences in the early treatment setting were reported. To date, OS results have been published for palbociclib, abemaciclib, and ribociclib [30,31,32,33,34,35], while those for dalpiciclib are yet to come. With a hazard ratio (HR) of 0.81 and a 95% confidence interval (CI) of 0.64–1.03 in the PALOMA-3 [31], and a HR of 0.96 and a 95% CI of 0.78–1.18 in the PALOMA-2 trial [30], palbociclib failed to show any OS benefit in both studies. While abemaciclib has already proven its efficacy regarding the OS in the MONARCH-2 study (HR 0.78; 95% CI 0.64–0.96) when being combined with fulvestrant in women who had not received chemotherapy and had a maximum of one prior ET for aBC [28], results from the MONARCH-3 trial in first-line patients are pending. For ribociclib, a consistent, statistically significant OS benefit could be shown in all three MonaLEEsa studies that is independent from the menopausal status or the ET partner (AI or fulvestrant) [32,33,34]. In the MonaLEEsa-2 trial, for instance, postmenopausal aBC patients treated with ribociclib and letrozole as first-line therapy achieved, with a median OS of 63.9 months, an OS prolongation of more than 12 months compared to the 51.4 months under endocrine monotherapy (HR 0.76; 95% CI 0.63–0.93) [32] (Table 1). It is of interest why these CDK4/6 inhibitors, despite being from the same drug family, lead to significantly different OS results. Potential reasons that are discussed are differences in the study designs and patient populations, but also in the substances’ pharmacology, affinity or in the binding to a specific side (more CDK4 than CDK6 and vice versa, for instance) [36].

### 2.3. CDK4/6i vs. Chemotherapy

The introduction of CDK4/6i led to a shift in the 1st and 2nd treatment lines of the therapeutic landscape. While, according to a German breast cancer registry, in 2015, almost 40% of the first-line patients received chemotherapy, this rate was significantly reduced by 2018 to 25% when all three inhibitors were available [37]. Three years later, in 2021, already, almost 75% of the first-line population was treated with a CDK4/6i and only 15% with chemotherapy [38]. This rapid implementation of CDK4/6i in the treatment of aBC caused further investigations regarding its comparability to chemotherapy. With regards to the PFS, it could be shown that no chemotherapy regimen with or without targeted therapy is significantly better than CDK4/6i in the 1st and 2nd treatment lines [39]. The above-mentioned German breast cancer registry could even demonstrate, in a recent analysis, that compared to patients treated with CDK4/6i or an ET monotherapy, those under chemotherapy in the first line had the most unfavorable prognosis regarding both the PFS and the OS. One possible reason for this outcome might also be that patients who are selected to receive chemotherapy as first-line treatment are those with a worse prognosis [38]. The PEARL trial was primarily designed to show the superiority of a palbociclib-based regimen compared to capecitabine. However, statistical significance could not be demonstrated, neither regarding the PFS nor the OS [40,41]. The RIGHT Choice study specifically analyzed the situation of pre- and perimenopausal women with aggressive disease, defined mostly by visceral metastases or rapid disease progression. In this patient population, it compared, as the first prospective trial, a ribociclib-based regimen to combinational chemotherapy in the first-line treatment setting. Ribociclib + ET could show a statistically significant PFS benefit of almost one year over chemotherapy (24.0 vs. 12.3 months; HR 0.54; 95% CI 0.36–0.79) [42]. On the basis of a better toxicity profile and quality of life (QoL) and at least similar or even better efficacy compared to chemotherapy, ET-based regimens in combination with CDK4/6i became the preferred treatment choice, even in patients with aggressive disease [12].

### 2.4. Resistance Mechanisms and Mutations

The question remains as to which resistance mechanisms lead to disease progression under CDK4/6i (Figure 1), how to treat these patients afterwards and whether a therapy with another CDK4/6i beyond progression makes sense. Novel treatment combinations will be discussed in Section 5. One study, however, that addressed the question on treatment beyond progression is MAINTAIN, a randomized, phase II trial of fulvestrant or exemestane, with or without ribociclib, after progression on CDK4/6i-based therapy in patients with aBC. Thus, 84% of the study population received palbociclib (*n* = 100), 11% ribociclib (*n* = 13) and 2% abemaciclib (*n* = 2) prior to the study treatment. Patients randomized to ribociclib plus fulvestrant or exemestane had, compared to those under ET without a CDK4/6i, a statistically significant PFS improvement (median PFS 5.33 months vs. 2.76 months; HR 0.59; 95% CI 0.38–0.91). At one year, 25% of the women on ribociclib + ET were still free from disease progression vs. only 7% of those on placebo + ET. Data on OS are pending [43]. This approach shows that even after disease progression on the first CDK4/6i, there is still some significant efficacy under a subsequent one. However, it is unclear whether this effect is restricted to the specific sequence of ribociclib being the second CDK4/6i. The phase III postMONARCH study (NCT05169567) is currently enrolling patients who progressed on a CDK4/6i, either in the adjuvant setting or as initial therapy for advanced disease, to be randomly assigned to fulvestrant plus abemaciclib or placebo [44]. The phase II PALMIRA study (NCT03809988) investigates the option of a palbociclib rechallenge in patients pre-treated with palbociclib [44].

Another trial found out that the ET partner for CDK4/6i also plays a significant role regarding the patients’ outcome. Women with aBC who were under an AI and palbociclib were screened in the PADA-1 study for a bESR1 mutation and then randomized 1:1 to either a continuation of the previous treatment with palbociclib plus the AI or to palbociclib plus fulvestrant instead of the AI. Median PFS from random assignment was 11.9 months in the palbociclib and fulvestrant group vs. 5.7 months in the palbociclib and AI group (HR 0.61; 95% CI 0.43–0.86). This way, PADA-1 was the first randomized prospective trial to show, in bESR1-mutated patients, that the type of ET a CDK4/6i is combined with has a relevant impact on the patients’ prognosis [45].

These examples demonstrate that further investigations are needed to better understand resistance mechanisms associated with the progression on ET in combination with CDK4/6i. Phase IV trials, such as CAPTOR (NCT05452213) with ribociclib or Minerva (NCT05362760) with abemaciclib, for instance, are designed to analyze biomarkers influencing the efficacy and resistance in aBC patients treated with each CDK4/6i [44].

## 3. Advancements in the Endocrine Treatment of Hormone Receptor-Positive, HER2-Negative Early-Stage Breast Cancer Patients

As mentioned above, ET consisting of Tamoxifen and AIs (+/− OFS) has been the standard of care in eBC patients for the past few decades. Mostly, a drug that is successful in the therapy of advanced disease is investigated in the early stage, too. Thus, due to the positive results in aBC, several studies analyzed the efficacy of CDK4/6i in eBC (Table 2).

### 3.1. Palbociclib Failing to Improve Invasive-Disease-Free Survival

As palbociclib was the first inhibitor from this family to be developed for the indication of metastatic BC, it was also the first one to be investigated in the early treatment setting. The multi-center phase III PALLAS trial enrolled 5796 patients with stage II and III disease to be randomly assigned to ET plus two years of additional palbociclib or ET alone. In the second planned interim analysis, no difference could be seen between the two treatment arms with regards to the 3-year invasive-disease-free survival (iDFS), so that the regimen was not recommended for this indication [49]. The results were confirmed by the final analysis at year four [47]. Another phase III study, Penelope-B, was investigated in parallel to the PALLAS palbociclib in patients with residual disease after neoadjuvant chemotherapy (NACT) and a high risk of recurrence defined by the CPS-EG score (clinical pathological staging-estrogen receptor grading score). In total, 1250 patients were randomized to ET plus either 13 cycles of palbociclib or placebo. However, similar to the PALLAS outcome, Penelope-B could not show any improvement in the iDFS in patients under additional palbociclib [48], making this CDK4/6i mainly a player in stage IV disease. Further studies with smaller sample sizes, such as the Appalaches (NCT03609047) comparing ET plus palbociclib to chemotherapy in elderly patients, the POLAR study (NCT03820830) investigating the efficacy of the same treatment combination in patients with isolated locoregional BC recurrence or the TRAK-ER (NCT04985266) treating ctDNA positive patients with palbociclib plus fulvestrant vs. standard ET, are ongoing [44].

### 3.2. Abemaciclib as the First New Drug in Two Decades to Complement Curative ET in Node-Positive Patients

Assuming, based on the exceptional OS improvement with ribociclib and abemaciclib in advanced disease, that the ET of the woman with eBC is also on the brink of a new era, this theory was first proven using abemaciclib. The monarchE, a multi-center randomized phase III trial, demonstrated, at an interim analysis in 5637 node-positive patients, a significant benefit of the addition of two years of abemaciclib to ET compared to ET alone. Further, 2-year iDFS rates were 92.2% vs. 88.7%, respectively (HR 0.75; 95% CI, 0.60–0.93) [50], resulting in an absolute delta of 3.5% between the study arms. As in other trials, such as Penelope-B, for instance, survival curves seemed to separate during the first few years but united at a later stage; further results from monarchE were awaited to see a clearer difference. At the 4-year analysis, the CDK4/6i again showed a better iDFS rate compared to the control arm (85.8% vs. 79.4%, respectively; HR 0.66; 95% CI 0.58–0.76) and the benefit even deepened over time, so that the absolute improvement grew to 6.4% [46]. Abemaciclib was approved by the FDA in 2021 in combination with ET for the therapy of node-positive patients with eBC and a high risk of recurrence [51]. A recent prespecified exploratory analysis from monarchE, looking mainly at patients who received NACT, could even extend the positive data situation for abemaciclib. Out of a total of 2056 node-positive patients pre-treated with NACT, the 2-year iDFS rate in the CDK4/6i arm was 6.6% better than in the control arm without the CDK4/6i (87.2% vs. 80.6%, respectively; HR 0.61; 95% CI 0.47–0.80), resulting in a 39% relative reduction in the risk of developing an iDFS event [52]. Thus, abemaciclib is not only the first CDK4/6i but, in general, the first drug in more than 20 years since the approval of AIs to be effective in hormone receptor-positive, HER2-negative eBC. While final OS results from the monarchE are pending, other trials that aim to analyze the role of abemaciclib in specific patient cohorts are ongoing. The ADAPTlate (NCT04565054), for instance, was designed to show whether abemaciclib added to an ongoing ET one to six years after BC diagnosis, i.e., “late”, is still effective. The POETIC-A (NCT04584853), however, is targeting postmenopausal women whose Ki-67 is persistently high after neoadjuvant ET, indicating endocrine resistance. In both trials, patients were randomized 1:1 to adjuvant ET alone or in combination with two years of abemaciclib [44].

### 3.3. Ribociclib with the Potential of Covering the Unmet Need in Stage II Disease

The third CDK4/6i ribociclib is also being investigated in the curative adjuvant setting within the multi-center phase III NATALEE (NCT03701334) trial. In total, 5101 patients with stage II and III disease were enrolled in the study to be randomly assigned to three years of ribociclib + ET vs. ET monotherapy [44]. In contrast to the above-mentioned trials with other CDK4/6i, ribociclib is not only used in a smaller dose in eBC than in aBC (400 mg vs. 600 mg, respectively) but is also a CDK4/6i that is combinable only with AI +/− OFS due to a prolongation of the QT interval when combined with tamoxifen [20]. However, a recent meta-analysis of 7030 premenopausal women from four randomized trials found out that, compared to tamoxifen + OFS, premenopausal women with a higher risk of recurrence have a better outcome under AI + OFS. The rate of BC recurrence was lower for women under an AI (rate ratio = RR 0.79; 95% CI 0.69–0.90) [53], so that the treatment combination from the NATALEE trial in stage II and III patients with an increased risk of recurrence seems feasible and logical. The main difference between the NATALEE and the monarchE trials is that, while the monarchE investigated only patients with axillary lymph node metastases, in case of positive study results from NATALEE, ribociclib could be used not only in node-positive but also in node-negative patients (partly with additional risk criteria), covering the currently unmet need in this population, too. Study results are expected to be presented in the near future. Meanwhile, the ADAPTcycle (NCT04055493) compares ET plus 600 mg of ribociclib to chemotherapy in women with an intermediate risk of recurrence according to the Oncotype DX recurrence score. It is one of few trials in the curative treatment setting comparing an ET-based regimen directly to chemotherapy [44].

### 3.4. CDK4/6i as Neoadjuvant Therapy

Some trials have investigated the role of CDK4/6i also in neoadjuvant therapy. In the single-arm NeoPalAna trial, patients with stage II and III BC received palbociclib plus anastrozole after four weeks of anastrozole monotherapy and underwent serial biopsies prior to breast surgery. The complete cell cycle arrest (CCCA) rate at C1D15 of palbociclib was significantly higher than under anastrozole alone at C1D1 (87% vs. 26%, respectively, *p* < 0.001) [54]. In the randomized phase II NeoPal study, 106 patients with stage II and III disease were enrolled, but this time, they were randomized to be treated with neoadjuvant palbociclib plus ET vs. chemotherapy. Both arms led to poor pathological complete response (pCR) rates (3.8% under ET + palbociclib and 5.9% under chemotherapy) and the study did not meet its primary endpoint [55]. Recently published survival outcomes did not differ between both arms, suggesting that a neoadjuvant letrozole-palbociclib strategy may allow chemotherapy to be spared in some patients [56]. Similar trials were performed with abemaciclib and ribociclib. In neoMonarch, patients treated with neoadjuvant abemaciclib achieved significant CCCA rates compared to those treated with anastrozole alone [57]. The phase II CORALLEEN trial compared six cycles of neoadjuvant letrozole and ribociclib to four cycles of chemotherapy and could show, with the help of PAM50 before–after analyses, that some patients with high-risk BC treated with ribociclib could achieve molecular downstaging at the time of surgery [58]. These results show that there is some potential for CDK4/6i also in the neoadjuvant treatment as it seems to have a certain impact on cell proliferation in eBC.

## 4. Impact on Patients’ Adherence and Quality of Life

No treatment is useful if patients’ adherence and QoL suffer significantly. It is wellknown, especially in the adjuvant ET setting, that adherence rates under AIs, for instance, decrease over the course of treatment, mainly due to adverse events (AEs) or certain characteristics [59,60]. However, despite having a life-threatening disease, even women with aBC terminate ET prematurely because of AEs [61]. As non-compliance and non-persistence are associated with a worse prognosis in BC patients [62] and any disease progression is, in turn, associated with a reduction in QoL [63], adherence and QoL under the combination of ET and CDK4/6i, that bring their own side effect profile with them, are of special interest.

The randomized trials in aBC have shown, across all CDK4/6i, that the QoL is either not significantly affected by the CDK4/6i or is even improved [64,65,66,67,68,69,70]. Analyses from the MonaLEEsa-2, -3 and -7 studies have, moreover, demonstrated that required dose modifications of ribociclib have no negative influence on survival outcomes [71,72]. Thus, doubts regarding patients’ outcome should not hinder physicians in reducing the medication in case of AEs, as the latter might result in patients’ non-persistence, leading to a worse prognosis.

Studies in eBC have further described patients’ adherence under CDK4/6i. In the PALLAS trial, 42.2% stopped palbociclib before two years of treatment were completed, out of which the majority, namely 27.2%, discontinued due to AEs. However, ET non-persistence rates did not differ between the two treatment arms [73]. Penelope-B confirmed discontinuation rates within one year of treatment with palbociclib. Overall, 17.5% terminated study treatment (3.0% because of AEs) and only 5.1% ET [48]. In the monarchE study, 25.8% of patients discontinued abemaciclib for reasons other than recurrence, including 18.5% due to AEs. Most of those who terminated CDK4/6i treatment continued receiving ET, while 6.5% discontinued both the CDK4/6i and the ET partner because of AEs. In the control arm, only 1.1% was non-persistent with ET [74], indicating that the combinational treatment seems to be associated with a higher risk of discontinuing the complete therapy. The dose-escalation study TRADE (NCT number not known at time of manuscript writing) will investigate the question of whether a titration of abemaciclib results in better adherence rates and less premature treatment discontinuations. Data from the NATALEE trial will reveal more about persistence rates and QoL outcomes under ribociclib in eBC setting.

**Figure 2:**
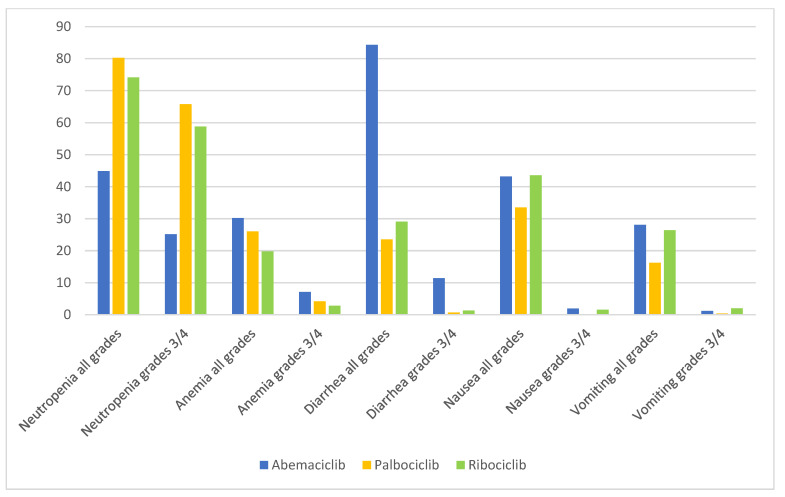
Relevant adverse events from phase III trials MONARCH-2 and -3 [23,24], PALOMA-2 and -3 [18,19] and MONALEESA-2, -3 and -7 [20,21,22] on average in percent.

When a decision is to be made between the substances, the treating physician must not only consider the survival and QoL data of each CDK4/6i but also the patients’ perspective of associated AEs. A survey among 209 oncologists and 304 patients was performed to see which AEs are key drivers for their therapy preferences. Among other risks, such as the risk of dose reduction due to AEs, risk of abdominal pain and the need for electrocardiogram monitoring, both groups rated risks of diarrhea (25% each) and grade 3/4 neutropenia (20% and 24%, respectively) as the most important attributes for treatment choice [75]. Figure 2 provides an overview of the most relevant AEs under the treatment with a CDK4/6i according to the phase III trials.

Despite ET adherence rates in need of improvement, in general, the treatment remains one of the best tolerable cancer therapies available. Still, the addition of a further substance such as the CDK4/6i to ET complicates patients’ adherence. Those at risk of early treatment discontinuation, e.g., because of deteriorating AEs, should, therefore, be more in focus to ensure timely side effect management, potential dose modification and patients’ compliance. Adherence programs in terms of digital health solutions might be one possible option to enable fast communication between the patient and the treating physician.

## 5. Modern Therapy Approaches and New Opportunities

### 5.1. The Role of HER2

HER2 positivity seems to be associated with higher levels of CDK4/6 activity, enabling response to CDK4/6i in this BC subtype [76]. In the primary preclinical cell culture experiments, a reasonable response to palbociclib was seen in both HER2-positive and hormone receptor-positive BC cell lines [17]. Several trials have, therefore, analyzed the role of CDK4/6i in HER2-positive BC. Early-phase studies with trastuzumab in combination with palbociclib or ribociclib, respectively, demonstrated, in general, a working treatment concept with good tolerability [77,78]. The MonarcHER, a phase II trial with a total of 237 patients, compared, in a three-arm design, a treatment with abemaciclib, trastuzumab and fulvestrant to abemaciclib with trastuzumab to standard-of-care chemotherapy with trastuzumab. The combination of abemaciclib, trastuzumab and fulvestrant significantly improved PFS compared to chemotherapy with trastuzumab (8.3 months and 5.7 months, respectively; HR 0.67; 95% CI 0.45–1.00), while there was no difference between abemaciclib with trastuzumab and chemotherapy with trastuzumab (HR 0.94; 95% CI 0.64–1.38) [79], also meaning that the ET backbone plays a significant role for the efficacy of a CDK4/6i.

Patients with HER2-positive aBC previously treated with trastuzumab and a taxane received, in further trials, T-DM1 combined with a CDK4/6i. Again, a good safety profile was seen, demonstrating that with an antibody–drug conjugate, even more aggressive treatment partners can be added to a CDK4/6i without safety concerns [80,81]. However, due to a fast-changing treatment landscape and extraordinary results from novel anti-HER2 therapies, such as trastuzumab-deruxtecan and tucatinib [82], late-phase trials with CDK4/6i for HER2-positive aBC are not being performed for every CDK4/6i. Currently, palbociclib and ribociclib are being investigated in triple-positive aBC within the phase III trials PATINA (NCT02947685), PATRICIA II (NCT02448420) and DETECT V/CHEVENDO (NCT02344472), respectively [44]. While results from the first two studies are expected to be presented in future, an interim analysis from the DETECT V study (triple-positive aBC patients randomized to trastuzumab + pertuzumab in combination with ribociclib + ET versus trastuzumab + pertuzumab in combination with chemotherapy followed by ribociclib + ET as maintenance treatment) was presented recently and showed no difference between a chemotherapy-containing and a chemotherapy-free regimen, neither regarding the PFS nor the OS. However, the tolerability was significantly better in the chemotherapy-free arm, so this phase III study is the first to demonstrate that CDK4/i—ribociclib in this case—in combination with antibodies is not inferior compared to chemotherapy and may, therefore, be an effective and safe treatment option for triple-positive BC patients [83].

Furthermore, since the introduction of multigene assays, it has been known that there is not only the BC subtype defined by immunohistochemistry (IHC) but also the one seen as the intrinsic subtype. Hence, intrinsic subtypes may differ from their immunohistochemical classification, which may also be associated with a switch in their risk categorization [84]. Luminal intrinsic subtypes have, in general, a better prognosis than HER2-enriched (HER2e) or basal-like ones, and it could be shown that, despite an immunohistochemically HER2-negative status, patients with intrinsic HER2e disease benefit from anti-HER2 treatments [85]. Therefore, it was again of special interest whether this also applies to a therapy with CDK4/6i, as these seem to have a certain efficacy in immunohistochemically HER2-positive BC, as mentioned above. To date, some studies have demonstrated efficacy of ribociclib in HER2e aBC patients, and not only across the MonaLEEsa study program [86] but also in a retrospective real-world analysis in comparison with palbo- and abemaciclib [87]. To further investigate the role of CDK4/6i in HER2e aBC, the ongoing randomized HARMONIA trial (NCT05207709) was set up and will analyze the efficacy of ribociclib versus palbociclib in this specific patient population [44]. Data from this study could help in defining the role of intrinsic subtypes for the treatment decision.

### 5.2. Novel Combination Partners

CDK4/6i have also been combined with novel substances from other drug families. Knowing that hormone receptor-positive, HER2-negative BC is generally a tumor with less immunoactivity, it is of interest whether the addition of checkpoint inhibitors, which usually trigger immune response, is feasible and effective in BC patients under CDK4/6i treatment. The phase II PACE trial randomized patients who progressed on CDK4/6i to fulvestrant +/− palbociclib +/− avelumab, an anti-PD-L1 antibody. While the 12-month PFS rates were 17.5% and 13.1% in the fulvestrant and fulvestrant + palbociclib arms, respectively, in the arm with the additional PD-L1 inhibitor avelumab, the rate was 35.6%. This resulted in an OS of 27.5 and 24.6 months in the fulvestrant and fulvestrant + palbociclib arms, and a total of 42.5 months in the fulvestrant + palbociclib + avelumab arm. Rates of immune-related toxicities under avelumab were low [88]. Other studies with abemaciclib and pembrolizumab, palbociclib and nivolumab, or ribociclib and spartalizumab demonstrated high grade 3 AE rates, especially for enhanced transaminases and inflammatory lung disease/pneumonitis, indicating that such combinations cannot be developed further. Thus, the PACE trial showed, for the first time in a CDK4/6i pre-treated population, a feasible therapy combination with a checkpoint inhibitor beyond progression on CDK4/6i.

Further combination partners are the novel group of oral SERDs as well as new SERMs. Several ongoing phase III studies, such as the SERENA-4 and -6 with camizestrant (NCT04711252 and NCT04964934), the persevERA with giredestrant (NCT04546009) and the EMBER-3 with imlunestrant (NCT04975308), evaluate the benefit of the according oral SERD in combination with CDK4/6i [44]. The ELAINE 2, a phase II study with the novel SERM lasofoxifene, could show, in combination with abemaciclib in patients, whose metastatic disease had progressed on hormonal therapy +/− CDK4/6i, a good safety profile and a certain efficacy, with a median PFS of 13.9 months [89]. The outcomes of late-phase trials will show whether these novel substances are effective combination partners for CDK4/6i.

### 5.3. Further CDK Inhibitors

There are also novel inhibitors of the CDK currently under development. Among others, with dalpiciclib, birociclib and lerociclib, there is a range of new CDK4/6i being evaluated in patients with hormone receptor-positive, HER2-negative aBC within phase III studies in China [44]. Trilaciclib, also a CDK4/6i, is even being investigated in patients with triple-negative BC, with promising results [90]. Dinaciclib, in contrast, inhibits the CDK1/2/5/9 and is also of interest for BC treatment [91].

All these advancements show that CDK’s role for the cell cycle is various and complex bearing high potential for further development.

## 6. Conclusions

The substance class of CDK4/6i has substantially improved the treatment landscape of hormone receptor-positive, HER2-negative BC patients. Achieving significant improvements in the survival rates in aBC patients and being the first substances in more than 20 years to improve DFS rates in eBC, CDK4/6i have set the new standard of care for patients suffering from this disease. The drugs have not only convinced researchers with better survival outcomes but also with manageable side effect profiles, as well as satisfying QoL data. Thanks to worldwide digitalization, nowadays, there is also more hope for better ET adherence rates using digital health solutions. For what happens after a CDK4/6i has been used, it remains important to better understand the mechanisms resulting in higher survival rates, but most of all, those that end in disease progression, to be able to develop novel substances on this basis. Further CDK inhibitors, treatment combinations with other drugs and different therapy sequences are under development, possibly leading to even more personalized BC treatment.

## Figures and Tables

**Table 1:** Efficacy of CDK4/6 inhibitors in hormone receptor-positive, HER2-negative aBC phase III trials (in alphabetical and numerical order; adapted and updated from [27]).

	Study Name	ET Partner	Sample Size	Rando-mization	Median PFS in Months (PFS = Primary Endpoint)					Median OS in Months (OS = Secondary Endpoint)				
					with CDK4/6 Inhibitor	without CDK4/6 Inhibitor	HR	95% CI	Statistically Significant as per Protocol	with CDK4/6 Inhibitor	without CDK4/6 Inhibitor	HR	95% CI	Statistically Significant as per Protocol
**ET +/− Abemaciclib**	MONARCH-2 [24,28]	Fulvestrant	669	2:1	16.4	9.3	0.55	0.45–0.68	yes	45.8	37.3	0.78	0.64–0.96	yes
	MONARCH-3 [23,29]	AI	493	2:1	28.2	14.8	0.54	0.42–0.70	yes	67.1	54.5	0.75	0.58–0.97	Final analys is not reported yet ^1^
**ET +/− Dalpiciclib**	DAWNA-1 [26]	Fulvestrant	361	2:1	13.6 ^2^	7.7 ^2^	0.45 ^2^	0.32–0.64 ^2^	yes ^2^	Final analys is not reported yet				
	DAWNA-2 [25]	AI	456	2:1	30.6	18.2	0.51	0.38–0.69	yes	Final analys is not reported yet				
**ET +/− Palbociclib**	PALOMA-2 [18,30]	AI	666	2:1	24.8	14.5	0.58	0.46–0.72	yes	53.9	51.2	0.96	0.78–1.18	no
	PALOMA-3 [19,31]	Fulvestrant	521	2:1	9.5	4.6	0.46	0.36–0.59	yes	34.9	28.0	0.81	0.64–1.03	no
**ET +/− Ribociclib**	MONALEESA-2 [22,32]	AI	668	1:1	25.3	16.0	0.57	0.46–0.70	yes	63.9	51.4	0.76	0.63–0.93	yes
	MONALEESA-3 [21,33]	Fulvestrant	726	2:1	20.5	12.8	0.59	0.48–0.73	yes	53.7	41.5	0.73	0.59–0.90	yes
	MONALEESA-7 [20,34]	OFS plus tamoxifen or AI	672	1:1	23.8	13.0	0.55	0.44–0.69	yes	58.7	48.0	0.76	0.61–0.96	yes

aBC: advanced breast cancer; AI: aromatase inhibitor; CI: confidence interval; ET: endocrine treatment; HR: hazard ratio; OFS: ovarian function suppression; OS: overall survival; PFS: progression-free survival; Tam: Tamoxifen. ^1^ An interim analysis has been reported [29]. ^2^ As assessed by an independent review committee.

**Table 2:** Efficacy of CDK4/6 inhibitors in hormone receptor-positive, HER2-negative eBC phase III trials at the latest analysis (in alphabetical order).

	Study Name	ET Partner	Sample Size	Rando-mization	Duration of CDK4/6 Inhibitor Therapy	DFS Rate					
						Latest Analysis	with CDK4/6 Inhibitor	without CDK4/6 Inhibitor	HR	95% CI	Statistically Significant as per Protocol
**ET +/− Abemaciclib**	monarchE [46]	AI or Tam +/− OFS	5637	1:1	2 years	year 4	85.8%	79.4%	0.66	0.58–0.76	yes
**ET +/− Dalpiciclib**	SHR6390-III-303 [44]	- ^1^	4350	1:1	- ^1^	not reported yet					
**ET +/− Palbociclib**	PALLAS [47]	AI or Tam +/− OFS	5796	1:1	2 years	year 4	84.2%	84.5%	0.96	0.81–1.14	no
	Penelope-B [48]	AI or Tam +/− OFS	1250	1:1	1 year	year 3	81.2%	77.7%	0.93	0.74–1.17	no
**ET +/− Ribociclib**	NATALEE	AI +/− OFS	5101	1:1	3 years	not reported yet					

AI: aromatase inhibitor; CI: confidence interval; DFS: disease-free survival; eBC: early breast cancer; ET: endocrine treatment; HR: hazard ratio; OFS: ovarian function suppression; Tam: Tamoxifen. ^1^ Data unknown at time of manuscript writing.

## Data Availability

Not applicable.
